# Home Range and Activity Patterns of Free-Ranging Cats: A Case Study from a Chinese University Campus

**DOI:** 10.3390/ani12091141

**Published:** 2022-04-28

**Authors:** Zhenwei Zhang, Yuhang Li, Sana Ullah, Lixin Chen, Sihan Ning, Liangyu Lu, Weiming Lin, Zhongqiu Li

**Affiliations:** 1 Lab of Animal Behavior & Conservation, School of Life Sciences, Nanjing University, Nanjing 210023, China; zhangzhenwei97@163.com (Z.Z.); 18687169197@163.com (Y.L.); 18246572625@163.com (L.C.); mg1930016@smail.nju.edu.cn (S.N.); 181840158@smail.nju.edu.cn (L.L.); lwmnju2018@163.com (W.L.); 2 Department of Zoology, Division of Science and Technology, University of Education, Lahore 54000, Pakistan; sunyuop@gmail.com

**Keywords:** free-ranging cats, home range, MCP, fixed kernel, activity patterns

## Abstract

**Simple Summary:**

In this study, we used collar-mounted sensors to determine the home range size of free-ranging cats on a Chinese university campus. Twenty-nine adult cats (fifteen males and fourteen females) were tracked via attached GPS units from October 2018 to June 2020. Throughout the study, home range sizes ranged from 0.56 to 19.83 ha at 95% KDE for all cats. The home range of free-ranging cats is affected by the breeding status and sex; for example, male cats tend to have a larger home range size in the breeding season than in the non-breeding season, and in the breeding season, male cats generally have a larger home range than females. In the study of activity patterns, we provided the activity steps of free-ranging cats at different times of the day, and the mean (±SE) number of steps a cat takes per day was 19,863.96 ± 1627.21. The results show that free-ranging cats have more intense activities at twilight and relatively lower activity intensity in the afternoon. Our study provided a case study of the home range and activity patterns of free-ranging cats living on a Chinese university campus, and provided theoretical support for the management and conservation implications of free-ranging cats in cities.

**Abstract:**

Human activities and the available resources influence the home range and activity patterns of free-ranging cats. Our objective in this study was to determine sex and breeding season vs. non-breeding season home range size, as well as activity patterns for unowned free-ranging cats at a university campus in China. Twenty-nine adult cats (fifteen males and fourteen females) were tracked with attached GPS units from October 2018 to June 2020. We considered the effects of sex and breeding status on the home range size of free-ranging cats. Male cats had larger home ranges (95% KDE: 12.60 ± 2.61 ha) than female cats (95% KDE: 5.02 ± 1.34 ha) in the breeding season. There was a seasonal effect on the home range size of male cats; for example, during the non-breeding season, the home range (95% KDE: 6.68 ± 1.22 ha) was smaller than that during the breeding season (95% KDE: 12.60 ± 2.61 ha), while female cats tended to have larger home ranges in the non-breeding season (95% KDE: 7.73 ± 2.77 ha) than in the breeding season (95% KDE: 5.02 ± 1.34 ha). We used the number of activity steps to measure the activity intensity of cats to explore their activity patterns. The mean (±SE) number of steps a cat takes per day was 19,863.96 ± 1627.21. There were two peak periods of activity in a day, 6:00–10:00 and 17:00–21:00. Our study provided a case study of the home range and activity patterns of free-ranging cats living on a Chinese university campus, and the results show that the home range of free-ranging cats is affected by the breeding status and sex, and free-ranging cats have more intense activities at twilight and relatively lower activity intensity in the afternoon. The results provided theoretical support for the management and conservation implications of free-ranging cats in cities.

## 1. Introduction

Domestic cats (*Felis silvestris catus*) are medium-sized carnivores and adaptable predators that are found worldwide due to their pet-like qualities and effectiveness as rodent predators [1]. However, they can have a major impact on native prey populations, including small mammals, avian species, and reptiles, and invasive cats on islands are responsible for 14% of global vertebrate extinctions [2,3,4]. Cats around the world have an ecological impact greater than native predators [5,6]. Free-ranging cats in high densities can potentially lead to devastating impacts on native animals [7,8]. In the case of high density, cat predation is a major cause of mortality for urban birds [9]. It is estimated that free-ranging domestic cats in China kill 26.9–55.2 billion birds per year, whereas 36.1–98 billion deaths per year are estimated for mammals [10]. As they are usually associated with human settlements and receiving food from humans, cats can affect native prey as well as native predators [11]. Free-ranging cats in cities sometimes become a public nuisance with urine and feces in the environment [12]. They can also transmit a variety of diseases to wildlife, pets, and humans, and spread toxoplasmosis (*Toxoplasma gondii*) through shed oocysts in feces [13]. Despite these harmful effects, management policies of free-ranging cat populations are primarily viewed as animal welfare issues rather than ecological issues [14].

The home range is an ecological concept in animal behavior and conservation biology research, and hence plays a key role in studying ecological processes such as habitat selection, animal social interactions, and evaluation of population density [15,16,17]. The accuracy of the estimation of animal homes affects the accuracy of our understanding of animal habits as well as the effectiveness of conservation and management [18,19]. There are many studies on home range sizes of feral cats and domestic cats outside China. In the past, the work of tracking home ranges and activities of cats has been carried out in various regions and countries around the world, such as in Hawaii [20,21], Australia [22,23,24], Europe [8,25], and Africa [3,26,27]. In previous studies, the home range sizes of cats differed due to their characteristics (e.g., owned and unowned cats) or reproductive status (e.g., sterilized or intact) and different habitat environments (e.g., island, town, farm, and nature reserve) [11,13,22,26,27,28,29,30,31]. Although there have been many studies on the effect of castration on home range sizes of cats [11,29], the impact of different breeding seasons on the home ranges is rarely discussed. Jeff et al. [13] failed to detect consistent differences in home range size between the sexes or among three seasons (summer, fall, and winter).

Activity patterns have been investigated in various mammals, but in domestic cats, the literature is scarce and contradictory [32]. Randall et al. [33] reported that cats do not exhibit circadian rhythms, and other reports described cats’ activity patterns as nocturnal or crepuscular [34]. Under the effects of different environmental variables, species may have different activity patterns. The simple diurnal–nocturnal dichotomy does not apply to all mammals, as the distinction between diurnal and nocturnal is not always clear [35]. Parker et al. [32] suggested that domestic cats have 24 h periodicity in locomotor and feeding rhythms, and locomotor behavior was more rhythmic than eating. In carnivores, it was shown that the activity patterns may be influenced by food availability and the activity cycle of prey [36]. Like many felids, cats are opportunistic hunters in the wild. This may indicate that the activity pattern of a cat is highly adjustable and adaptable. Studies have pointed out that different housing conditions influence the activity patterns of domestic cats, cats that live in strong symbiosis with owners, with respect to the cats living in yards, the amount of activity that was higher during the photophase [35], and the care provided by the cat owner influences the cat’s impact on its environment [37]. Feeding can facilitate the establishment of a relationship between a cat and a person [38].

From domestic cats that are dependent on humans for food and shelter to feral cats that are fully independent of humans, the cat–human relationship has evolved into a variety of forms [37]. Free-ranging cats can be distinguished from domestic and feral cats by having uncontrolled movement and partly controlled resource provisioning [39]. Many studies have been carried out on the home range size and movement of free-ranging cats in various areas of the world; however, less work has been performed within China, particularly in the relatively special environment of the university campus. Unlike ordinary communities, Chinese universities are closed and independent, with a high population density of cats on campus and frequent feeding of cats. The study of free-ranging animals improves the knowledge about the possible negative impact of pets left unattended outdoors on endemic species of birds, mammals, and reptiles, and it is important to understand free-ranging cats’ home range and factors that affect their tenancy. Our objectives were to understand the home range size and activity patterns of free-ranging cats at a Chinese university campus. We evaluated the effect of sex and breeding status on the home ranges, and tested the hypothesis that male cats tend to be more territorial than female cats, especially during the breeding season. We also tried to describe the activity pattern of free-ranging cats using the number of activity steps in this study.

## 2. Materials and Methods

### 2.1. Study Area

The study was conducted at the Xianlin Campus (32°07′ N, 118°57′ E) of Nanjing University, located in Northeast Nanjing, Jiangsu Province, China. The campus consists of approximately 253 ha of land and approximately 120 ha of construction area, with approximately 30,000 teachers and students on the campus. The topography consists of low rolling hills, as well as bush and open terrain. There is a small stream on the campus, approximately 1.2 km-long and approximately 10 m-wide, with riparian cover along the stream. There is a subtropical humid climate with four distinct seasons and abundant rain. The average annual rainfall per year is 117 days, with an average rainfall of 1106.5 mm, the relative humidity is 76%, and the frost-free period is approximately 237 days. From late June to early July every year is the rainy season. The annual average temperature is 15.4 °C, and the annual extreme temperature is highest at 39.7 °C and lowest at −13.1 °C (China Meteorological Administration).

### 2.2. GPS Tracking

All cats involved in the study were unowned and lived on Xianlin Campus, and were fed by the teachers and students spontaneously. These cats were considered to be free-ranging as they did not enter human dwellings but were fed sporadically by people. This study used GPS positioning collars from October 2018 to June 2020 to track and record the activity trajectories of 29 free-ranging cats on the campus, each recorded for 5 days. Domestic cats are animals that breed seasonally, and spring is the period of a high incidence of estrus and mating. We consider May and June in late spring and early summer as the breeding seasons for free-ranging cats, and October, November, and December as non-breeding seasons in autumn and early winter. The GPS positioning collar sent one positioning point every hour, and 24 locations per day were taken, for a total of 120 locations per cat. During the research, the monitoring time for each cat was five days because it was limited by the battery capacity of the GPS positioning collar. Kays et al. [7] evaluated the change in home-range size with increased tracking duration, and the results showed that the variance decreased sharply at five days. The living environment of cats was relatively stable, so we considered it reasonable to use five days to measure the home range size. When calculating the activity steps of cats, we selected the activity intensity information of ten cats because the previous tracking data did not save the activity intensity information. The specific information of these cats is shown in Supplementary Table S2.

Home range and locomotor activity research used a Pawfit-S (Latsen Technology Ltd., London, UK) pet positioning collar as a GPS (Global Positioning System) radio device (49 × 36 × 13 mm), of 29.5 g in weight. Pawfit-S uses an accelerometer that is set up to record the integration of intensity, amount, and duration of movement in all directions. The collar adopts unique data processing and calculation methods specially designed for pets, similar to a health monitoring device used by people, and it can record the activity intensity of animals in each hour and the total number of steps in each day. The Pawfit application 1.4.2 (Latsen Technology Ltd., London, UK) recorded the location of the cats. The location was exported and saved in latitude and longitude coordinate formats, and ArcGIS was used to calculate the home range.

### 2.3. Monitoring Potential Ethical Issues in Collared Cats

The weight of the positioning device is also a factor that must be considered. Some studies have shown that devices weighing less than 3% of the body mass of animals may still have impacts, so the weight should be no more than 2% body mass (BM), and a heavier device will make the cat’s home range size smaller and bring safety risks [40,41]. Our device in this study met this standard and weighed less than 30 g. To prevent animals from being harmed by positioning collars, as mentioned in some studies [42,43], we regularly observed the collar-worn cats under our study on a daily basis to monitor potential health problems.

We did not use anesthesia on the cats in this study—we minimized the stress by feeding them cat food and petting them during capture and handling. The captured individuals were adults, and while sampling/capturing, no individuals were injured or died. The cats were closely tracked in the first 24 h to ensure that collars did not impede movement, interfere with behaviors, or cause other safety issues. The collars were fitted correctly to make sure there was no risk of getting paws caught in the collar, and no rubbing of the neck. If any animal was distressed, we would find it by locating it and use food to lure it and take off the collars immediately, and at the end of the study, the collars were all removed.

### 2.4. Statistical Analyses

We used Home Range Tools (HRT) in ESRI ArcMap (Version 10.3) to calculate the 100% Minimum Convex Polygon (MCP) for each cat. We used “fixed mean” to calculate the arithmetic mean of all x (longitude) and y (latitude) coordinates and then selected the requested percentage of points closest to that arithmetic mean point. We also used HRT to calculate home range areas (95% and 50% probability areas) using a fixed kernel estimator (KDE) (the plug-in estimator was used to estimate a kernel smoothing parameter for each cat and a cell size of 10) for comparison to previous studies, as both MCP and KDE are commonly reported. Each free-ranging cat’s total range of activities was recorded, calculated, and counted within five days. Data analyses were performed using the statistical software SPSS. We used a two-way analysis of variance (ANOVA) to determine the effects of sex, breeding status, and their interaction on the home range size. The Shapiro–Wilks test was used to evaluate the normal distribution of the residuals. The home ranges were calculated based on two periods: breeding season (May 2019; June 2020) and non-breeding season (October to November 2018; December 2019).

The Pawfit application 1.4.2 (Latsen Technology Ltd., London, UK) provides an exercise interface, which shows the number of pet steps in a day and the activity intensity of each hour in a day. Activity intensity is displayed as a bar chart with 24 columns, and each column represents the intensity of activity within an hour. We used Image-Pro Plus 6.0 to count the number of pixels per column. We divided the number of steps per day by the total number of pixels per day and multiplied this by the number of pixels per hour to obtain the number of active steps per hour. All datasets are presented as the mean ± SE (standard error), and differences between means were considered significant at *p* < 0.05.

## 3. Results

### 3.1. Home Ranges

Home range estimates for 29 collared free-ranging cats (15 males, 14 females) from Xianlin Campus of Nanjing University were determined for the study period between October 2018 and June 2020. In the breeding season, a total of 14 (6 males, 8 females) cats were captured and recorded; in the non-breeding season, 15 (9 males, 6 females) cats were captured and recorded. A total of 3480 GPS locations were recorded during the duration of the study for all collared cats, and each cat had 120 locations.

Throughout the study, home range sizes ranged from 1.23 to 16.93 ha at 100% MCP for all cats, 0.56 to 19.83 ha at 95% KDE, and 0.08 to 5.16 ha at 50% KDE. The mean home range size for all free-ranging cats using 95% KDE was 7.66 ± 1.01 ha (100% MCP was 6.80 ± 0.81 ha). The mean core range size for all free-ranging cats using 50% KDE was 1.5 ± 0.26 ha (Table S1). Based on the 95% KDE method, when two-way ANOVA was used, there was an interaction between sex and season in the influence of the home range: F_1,25_ = 5.11, *p* = 0.03, and partial Eta square = 0.17. Pairwise comparisons were used to analyze the individual effect results for each category.

**Figure 1:**
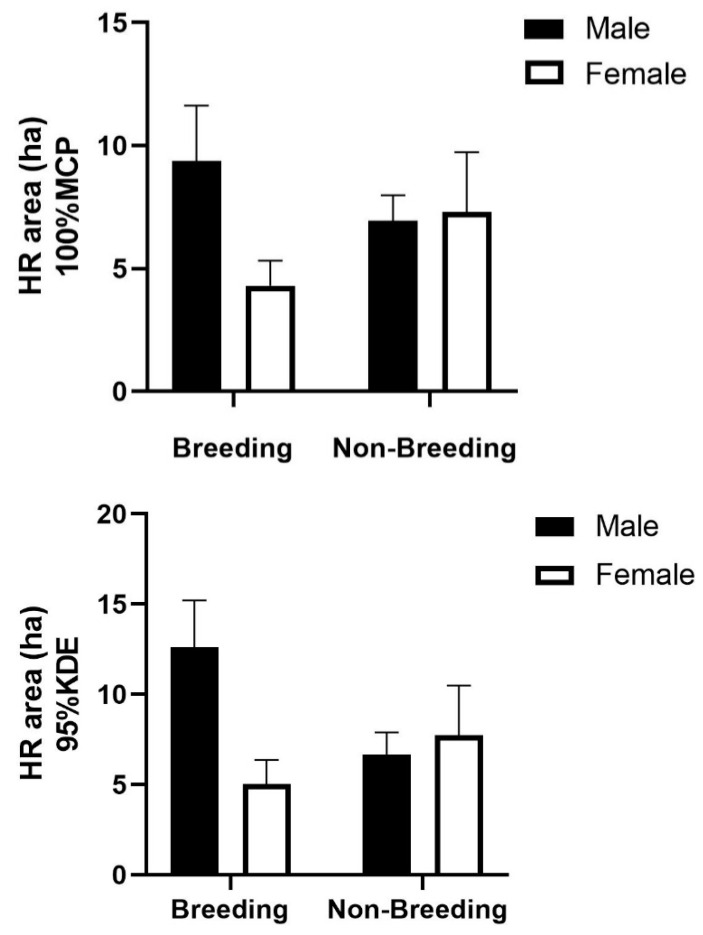

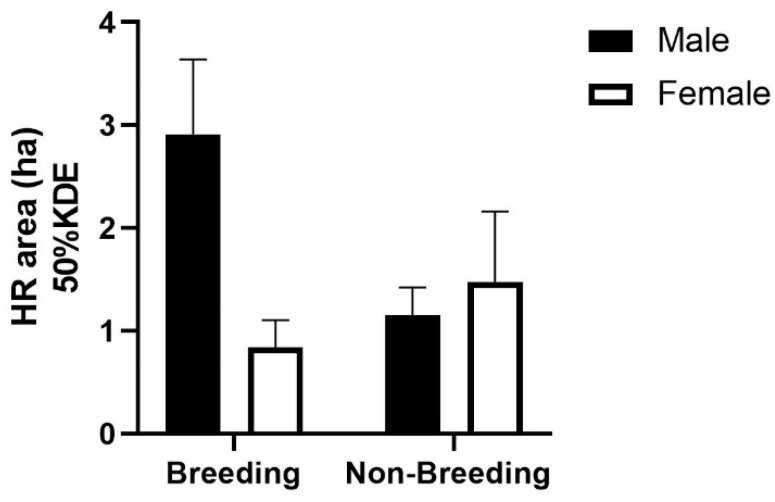
Breeding season and non-breeding home ranges for male and female free-ranging cats at Nanjing University (Xianlin Campus). KDE (fixed kernel estimator).

There were significant differences between breeding season and non-breeding season 95% KDE home ranges of male cats at the Xianlin campus (F_1,25_ = 4.93, *p* < 0.05). Male cats’ home ranges in the breeding season (12.60 ± 2.61 ha) were 5.93 ha (95% CI: 0.43–11.42) larger than in the non-breeding season (6.68 ± 1.22 ha). Although home ranges used by females in the non-breeding season (7.73 ± 2.77 ha) were generally larger than in the breeding season (5.02 ± 1.34 ha), there were no significant differences between the seasons (F_1,25_ = 0.98, *p* = 0.33). There were significant differences between male and female 95% KDE home ranges of free-ranging cats in the breeding season (F_1,25_ = 7.69, *p* = 0.01), while the differences were not significant in the non-breeding season (F_1,25_ = 0.16, *p* = 0.70). In the breeding season, the home range sizes of the male cats were 7.58 ha (95% CI: 1.95–13.21) larger than those of female cats (Figure 1, Table 1).

### 3.2. Activity Patterns

**Figure 2:**
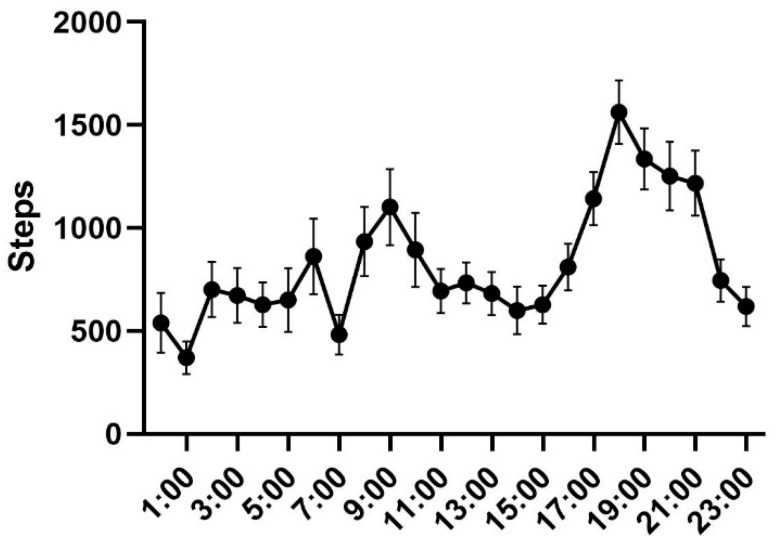
The activity patterns of cats’ locomotory activity. The value in the figure is the average number of steps taken by 10 cats for 50 days in each time period of the day.

Our positioning collar recorded not only the activity position of cats but also their activity intensity at each time of the day (indicated by the number of exercise steps). We used the activity data of 10 free-ranging cats (5 M, 5 F), each of which was tracked for 5 days, for a total of 50 days to calculate their activity patterns. There were two peak periods of activity in a day, 6:00–10:00 and 17:00–21:00. It can be seen that the activity intensity of free-ranging cats was low in the afternoon and evening (Figure 2). The highest value of active steps was 1563 at 18:00, and the lowest value was 371 at 1:00. The mean (±SE) number of steps a cat takes per day was 19,863.96 ± 1627.21 (Table S2).

## 4. Discussion

In this study, we described the home range size and activity patterns of unowned, free-ranging cats at the Xianlin campus of Nanjing University. The mean home range sizes (7.66 ha 95% KDE) and core sizes (1.50 ha 50% KDE) in this study were relatively small compared with previous studies. In previous studies, the home range of feral cats was as high as hundreds of hectares [24,25,44,45], while owned cats (farm cats and domestic cats) were much smaller, less than 10 hectares [11,13,27,46]. This concentrated home range is the same as that found by Genovesi, Besa et al. [47], who observed that cats have a small range of activities in areas with high population density in cities, while they have larger home ranges in the wilderness. Generally, home range size is influenced by resource availability and food abundance. In this study, the small home ranges may indicate that food provided by humans affects the activities of free-ranging cats, as they can obtain food from students on campus, instead of hunting for food in larger home ranges. Our study indicated that male cats tended to have a larger home range in the breeding season than in the non-breeding season, while we found the exact opposite results in female cats. We also found that male cats tended to have a larger range than female cats in the breeding season, which was similar to several other studies [24,29,44,48,49]. We speculate that this may be related to the courtship and territory behavior of male cats.

Our research site was on campus, and due to the special environment of Chinese university campuses, the population density of cats in the research area was relatively high. Students on campus often feed and pet cats, and the cats on the campus are less aggressive than feral cats. Although there have been many studies on the home range, few studies have explored the activity patterns of cats. The monitoring of activity requires more sophisticated instruments. In this study, we provided activity patterns of free-ranging cats using movement steps as a measurement parameter, which may be a more accurate measure. Our research has found that cats may have more intense activities at twilight and relatively lower activity intensity in the afternoon. They also had more activity around sunrise, which is similar to some previous studies [47,50]. This result suggests that trapping for management of the population should therefore yield the highest number of captures at twilight and sunrise, when cats are more active [51].

However, the population of cats in the study area varied greatly, and the cats caught in the previous season often disappeared in the next season. It was difficult for us to find the cats caught in the previous season. Since we used different individuals in different breeding seasons, our conclusions need to be interpreted with caution, especially since the results show that home ranges of cats varied greatly between individuals, and it is possible that this difference may also be caused by individuals. In this study, we provided the home ranges of free-ranging cats that were tracked for five days. Although the living environment of the cats was relatively stable in this study, and some other studies [7] have tracked home ranges for five days, to understand the migration and more precise home ranges of free-ranging cats, long-term tracking is necessary in the future.

Despite these limitations, we have presented a case study on free-ranging cats’ home ranges and activity patterns, and provided basic biological information about the free-ranging cats on a Chinese university campus. Unfortunately, the sample size of cats in this study was relatively small, and we look forward to a better experimental design and a larger amount of data to verify this conclusion in the future.

## 5. Conclusions

Our study described home ranges and activity patterns of free-ranging cats on a Chinese university campus, and the results show that male cats tended to have a larger home range size in the breeding season. Free-ranging cats showed increased activity with rhythms of behavior, and they showed more intense activities at twilight and a relatively lower activity intensity in the afternoon.

## Figures and Tables

**Table 1:** Home range estimates (ha; 100% MCP (Minimum Convex Polygon), 95% KDE (fixed kernel estimator), and 50% KDE) for free-ranging cats at Nanjing University (Xianlin Campus) across breeding and non-breeding seasons (Lowercase letters are test results, and different letters in the same row represent significant differences; SE (standard error)).

Sex	Season	*n*	100% MCP		95% KDE		50% KDE	
			Mean	SE	Mean	SE	Mean	SE
Male	Non-Breeding Season	9	6.96 ^ab^	1.03	6.68 ^b^	1.22	1.15 ^b^	0.27
	Breeding Season	6	9.37 ^a^	2.25	12.60 ^a^	2.61	2.91 ^a^	0.73
Female	Non-Breeding Season	6	7.30 ^ab^	2.43	7.73 ^ab^	2.77	1.47 ^ab^	0.69
	Breeding Season	8	4.31 ^b^	1.01	5.02 ^b^	1.34	0.84 ^b^	0.26

## Data Availability

Not applicable.

## Supplementary Materials

The following supporting information can be downloaded at https://www.mdpi.com/article/10.3390/ani12091141/s1, Table S1: Sex and captured time of cats used to determine home ranges on the Nanjing University Xianlin campus (2018–2020). Table S2: Activity and steps data of free-ranging cats over different time periods in one day (The data in the table is the average number of activity steps of an individual in the same time period within five days).
