# Supplementary material for: Home Range and Activity Patterns of Free-Ranging Cats: A Case Study from a Chinese University Campus
Source: Animals (Basel). 2022 Apr 28;12(9):1141. doi: 10.3390/ani12091141 (PMC9103849; doi:10.3390/ani12091141)
Supplement: Supplementary file 1 [file animals-12-01141-s001.zip › animals-1568092-supplementary/Supplementary Materials.pdf]

Table S1. Sex, Captured time of cats used to determine home range on the Nanjing University Xianlin campus (2018--2020).

Cat name	Sex	Date	Season	100%MCP (ha)	95%KDE (ha)	50%KDE (ha)
FM1	Female	October 2018	Non-Breeding	9.35	10.19	1.60
FM2	Female	October 2018	Non-Breeding	9.42	10.14	1.34
FM4	Female	October 2018	Non-Breeding	16.93	18.78	4.70
FM5	Female	November 2018	Non-Breeding	5.52	5.06	0.85
FM6	Female	November 2018	Non-Breeding	1.35	1.63	0.26
I-5F	Female	December 2019	Non-Breeding	1.23	0.56	0.08
FMA	Female	May 2019	Breeding	7.60	12.21	2.51
FMB	Female	May 2019	Breeding	6.87	6.07	0.84
FMC	Female	May 2019	Breeding	8.56	8.87	1.18
FMD	Female	May 2019	Breeding	2.57	3.22	0.73
FME	Female	May 2019	Breeding	1.96	2.21	0.25
III-2F	Female	June 2020	Breeding	2.95	2.94	0.46
III-3F	Female	June 2020	Breeding	2.47	3.42	0.55
III-4F	Female	June 2020	Breeding	1.48	1.22	0.21
I-2M	Male	December 2019	Non-Breeding	5.15	2.16	0.19
II-10M	Male	December 2019	Non-Breeding	8.78	7.46	1.00
II-11M	Male	December 2019	Non-Breeding	7.15	4.25	0.31
II-12M	Male	December 2019	Non-Breeding	5.01	4.79	0.79
M3	Male	November 2018	Non-Breeding	5.44	6.40	1.28
M4	Male	November 2018	Non-Breeding	13.00	12.37	2.04
M5	Male	November 2018	Non-Breeding	5.28	6.29	1.07
M6	Male	November 2018	Non-Breeding	9.85	12.60	2.79
M7	Male	November 2018	Non-Breeding	2.95	3.77	0.88
MA	Male	May 2019	Breeding	15.29	18.24	5.05
MB	Male	May 2019	Breeding	14.71	16.12	2.53
MC	Male	May 2019	Breeding	3.30	4.63	1.04
MD	Male	May 2019	Breeding	4.30	6.30	1.39
ME	Male	May 2019	Breeding	12.95	19.83	5.16
III-1M	Male	June 2020	Breeding	5.68	10.49	2.29
